# 

**Published:** 1883-11

**Authors:** 


CONTENTS.

| | Page. | | Page. |
|---|---|---|---|
| Subscriptions and Premiums | 223 | The Rural Masses of China | 231 |
| The Object of Exercise | 224 | American Pharmacy Abroad | 232 |
| Bad Air | 225 | Weakly Children | 232 |
| Colds | 226 | British Pastry | 234 |
| Nutriment | 227 | When to take Medicine | 236 |
| Philosophy of Eating | 228 | The Weight of Great Men | 237 |
| Fig Paste | 230 | Food Adulteration | 238 |

